# Survival Outcomes and Prognostic Factors in Glioblastoma

**DOI:** 10.3390/cancers14133161

**Published:** 2022-06-28

**Authors:** Nicholas F. Brown, Diego Ottaviani, John Tazare, John Gregson, Neil Kitchen, Sebastian Brandner, Naomi Fersht, Paul Mulholland

**Affiliations:** 1 Department of Oncology, University College London Hospitals, London NW1 2PG, UK; n.brown4@nhs.net (N.F.B.); d.ottaviani@ucl.ac.uk (D.O.); naomi.fersht@nhs.net (N.F.); 2 UCL Cancer Institute, University College London, London WC1E 6DD, UK; 3 Department of Statistics, London School of Hygiene and Tropical Medicine, London WC1E 7HT, UK; john.tazare1@lshtm.ac.uk (J.T.); john.gregson@lshtm.ac.uk (J.G.); 4 Department of Neurosurgery, National Hospital for Neurology & Neurosurgery, Queen Square, London WC1N 3BG, UK; neilkitchen@nhs.net; 5 Division of Neuropathology, National Hospital for Neurology & Neurosurgery, Queen Square, London WC1N 3BG, UK; s.brandner@ucl.ac.uk; 6 Department of Neurodegenerative Disease, UCL Queen Square Institute of Neurology, Queen Square, London WC1N 3BG, UK

**Keywords:** glioblastoma, MGMT, IDH, biomarker

## Abstract

**Simple Summary:**

Glioblastoma is the most common tumour that originates in the brain in adults. Most of the published data on glioblastoma are from patients in clinical trials who tend to be younger and fitter than the average patient. We therefore looked at patient demographic, tumour characteristics, and treatments received in a group of 490 real-world patients with glioblastoma to evaluate their survival, and to investigate whether we could find any factors that were associated with longer survival. Overall, the average survival of patients was 9 months. Patients tended to live longer if they were younger, had surgery, if they had further treatment after surgery (chemo- or radio-therapy), or if they had a tumour marker called MGMT promotor methylation.

**Abstract:**

Background: IDH-wildtype glioblastoma is the most common malignant primary brain tumour in adults. As there is limited information on prognostic factors outside of clinical trials; thus, we conducted a retrospective study to characterise the glioblastoma population at our centre. Methods: Demographic, tumour molecular profiles, treatment, and survival data were collated for patients diagnosed with glioblastoma at our centre between July 2011 and December 2015. We used multivariate proportional hazard model associations with survival. Results: 490 patients were included; 60% had debulking surgery and 40% biopsy only. Subsequently, 56% had standard chemoradiotherapy, 25% had non-standard chemo/radio-therapy, and 19% had no further treatment. Overall survival was 9.2 months. In the multivariate analysis, longer survival was associated with debulking surgery vs. biopsy alone (14.9 vs. 8 months) (HR 0.54 [95% CI 0.41–0.70]), subsequent treatment after diagnosis (HR 0.12 [0.08–0.16]) (standard chemoradiotherapy [16.9 months] vs. non-standard regimens [9.2 months] vs. none [2.0 months]), tumour MGMT promotor methylation (HR 0.71 [0.58–0.87]), and younger age (hazard ratio vs. age < 50: 1.70 [1.26–2.30] for ages 50–59; 3.53 [2.65–4.70] for ages 60–69; 4.82 [3.54–6.56] for ages 70+). Conclusions: The median survival for patients with glioblastoma is less than a year. Younger age, debulking surgery, treatment with chemoradiotherapy, and MGMT promotor methylation are independently associated with longer survival.

## 1. Introduction

Glioblastoma is the most common malignant primary brain tumour in adults with an incidence of 3–4/100,000, and accounting for approximately half of all malignant primary brain tumours [1,2]. An initial diagnosis is typically made on the histology from tissue taken at surgical resection or stereotactic tumour biopsy. Diagnosis solely on the basis of imaging may occur if the risk of biopsy is too high, or if treatment is not contemplated-usually due to frailty [3]. Overall, over 90% of patients have a histological diagnosis, but this is less than 60% in those over 70 years old [4].

Standard treatment consists of surgical resection if possible, followed by radiotherapy and temozolomide chemotherapy, where, 60 Gray (Gy) of focal radiotherapy is administered in 2 Gy fractions. Temozolomide is given concurrently alongside radiotherapy and then for a further six months. Hypofractionated radiotherapy may be used in those patients not expected to tolerate standard radiotherapy [3,5]. Recently, the addition to standard therapy of tumour treating fields following radiotherapy has shown improved outcomes [6]. At relapse, typically, nitrosurea-based chemotherapy is given, although no therapies at relapse have demonstrated survival benefit in clinical trials.

Survival outcomes are bleak with a median survival in registry databases of 6–10 months [2,4] and 14.6–21.1 months in those treated with standard therapy in clinical trials [7,8,9,10,11,12,13]. Several demographic and molecular prognostic factors are recognised. However, there is limited information on the application of prognostic factors outside of the highly selected population within clinical trials, particularly in the temozolomide era, and there are few studies that include both molecular and clinical factors. We characterised the glioblastoma population treated at our centre, to determine the prognostic factors that can be identified at the time of diagnosis, and to assess the role of subsequent treatments.

## 2. Materials and Methods

### 2.1. Patients

All patients with primary glioblastoma that was histologically diagnosed using the 2007 WHO Classification of CNS Tumours (but with comprehensive molecular workup equivalent to glioblastoma IDH- (isocitrate dehydrogenase) mutant/IDH-wildtype in the 2016 Classification, and to Glioblastoma, IDH-wildtype/Grade 4 Astrocytoma, IDH-mutant within the recent 2021 WHO Classification) at our centre between January 2011 and December 2015 were identified using a pathology database [1,14,15,16]. Patients were excluded if they were not UK residents or if they had a previously identified primary brain tumour (either histologically or radiologically diagnosed). Patient demographics, treatment received, and date of death or last contact were collated from the Electronic Patient Record (EPR) at University College London Hospitals; 90% of patients of patients had died at the time of analysis, suggesting adequate capture of patient death records. The tumour molecular characteristics were collated via the centre’s pathology database (*IDH1* (R132H) mutation determined by immunohistochemistry; *MGMT* (O^6^-methylguanine-DNA-methyltransferasepromotor) methylation by methylation sensitive high resolution melting analysis; and Sanger sequencing for mutation hotspots in *IDH1* and *IDH2*, copy number assays for the *PTEN* (phosphatase and tensin homolog) locus on chromosome 10, *EGFR* (epidermal growth factor receptor locus on chromosome 7, and chromosomal arms 1p and 19q by quantitative PCR [15]. Survival was defined as time from the date of diagnosis to death or last known contact.

### 2.2. Statistical Analysis

We performed two main sets of analyses. First, we investigated the impact of age, gender, and tumour biomarkers on survival in the full cohort (*n* = 490). We used Kaplan−Meier survival curves and estimates of median survival to describe univariate associations with survival. To investigate multivariate predictors of survival, we built a Cox proportional hazard models to predict survival, including age, gender, and all tumour biomarkers as candidate predictors. We used a forward stepwise variable selection with a *p*-value for inclusion of 0.05. To allow for estimation in the presence of missing data, we performed multiple imputation with chained equations. The imputation model included all candidate predictors, the outcome, and the cumulative hazard functions [17]. We used 10 imputed datasets.

Second, we described the association of treatment with survival. We restricted these analyses to patients in whom treatment was known. In standard practice, adjuvant chemoradiotherapy is initiated within six weeks of diagnosis; we were therefore unable to reliably define the treatment choice for each patient until this time because of our retrospective study design. We therefore included only patients surviving for a minimum of 6-weeks in these analyses (although sensitivity analyses including the first 6 weeks gave similar findings). We investigated the effect of treatment on survival using Cox model adjusted biomarkers found to be associated with outcome in the full patient cohort. We additionally tested for evidence that tumour biomarkers modified the effect of treatment using formal tests of interaction. Statistical analyses were performed using Stata 14.2 (Stata Corporation, College Station, TX, USA).

## 3. Results

### 3.1. Patients

In total, 517 consecutively diagnosed patients with primary IDH-wildtype glioblastoma or Grade 4 IDH-mutant astrocytoma were identified; 27 patients who were not UK residents were excluded from all of the analyses, leaving a total of 490 patients. The median age at baseline was 59 years (Supplementary Materials Figure S1), 293 (59.8%) were male and 197 (40.2%) female, 51 (11% of 482 patients with available data) had *IDH1* or *IDH2* mutations detected in their tumours, and 234 (51% of 456) of patients had methylation of the *MGMT* promotor. Patient characteristics are shown in Table 1.

### 3.2. Treatment

**Figure 1:**
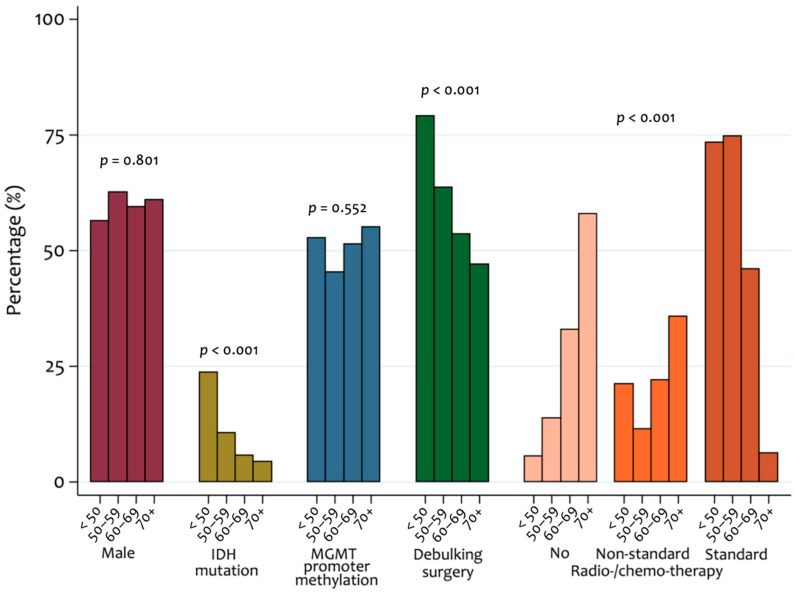
Patient characteristics by age category. *p*-values indicate the significance of observed differences between age groups for each variable.

It was found that 60% of patients had a primary debulking surgery and 40% of patients had biopsy only. Patients who had debulking surgery were younger than those who had biopsy only (median age 56 vs. 63 years, *p* < 0.001) (Figure 1 and Table S1). Thirty-six patients (7%) who died within six weeks of diagnosis were not included in the analyses of subsequent treatments. Subsequent treatment details were known in 314 patients (69%); where subsequent patient treatment was unknown, it was usually because patients were referred to other centres. Among these patients, 254 (81%) had further active therapy. Patients who had further active therapy tended to be younger (median age 58.8 years vs. 68.8, *p* < 0.001) and more frequently had debulking surgery (69% vs. 31%, *p* < 0.001) (Figure 1 and Table S1).

In those who had adjuvant therapy, 176 (69%) had radical radiotherapy (RT) (60–65 Gray in 30 fractions) with temozolomide (TMZ) chemotherapy (standard therapy), 43 (17%) had short course RT alone, 20 (8%) had radical RT alone, 10 (4%) had short course RT with TMZ, and 3 (1%) had TMZ alone. Patients receiving standard care tended to be younger than those receiving other active (non-standard) therapies (52.2 years vs. 60.2 years, *p* < 0.0001) (Figure 1 and Table S1).

At relapse, 57 (22%) of patients who had treatment after initial diagnosis had further surgery, 94 (37%) had second line chemotherapy, and 29 (11%) had third line chemotherapy. Of those who received chemotherapy in the relapsed setting, PCV (procarbazine, lomustine, vincristine) or lomustine monotherapy were most frequently used (in 71, 76% of patients), followed by bevacizumab (28, 30%), immune checkpoint inhibitors (20, 21%), temozolomide (7, 7%), carboplatin (7, 7%), or other agents (9, 10%).

### 3.3. Survival Outcomes

**Figure 2:**
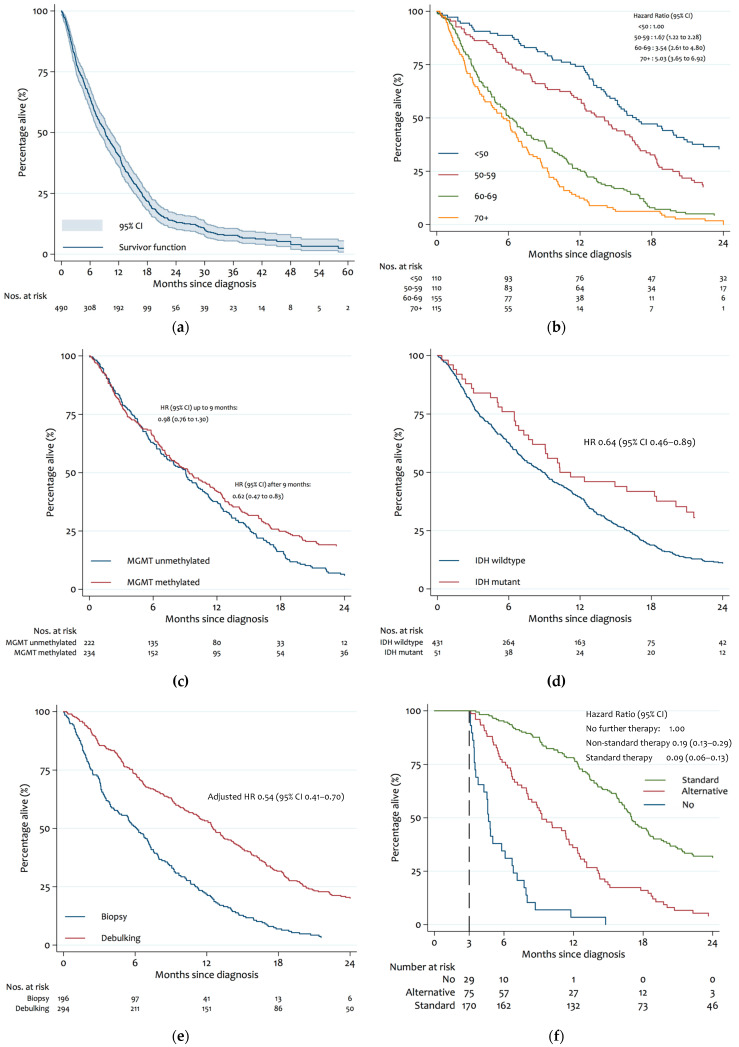
Kaplan−Meier survival estimates: (**a**) overall survival; (**b**) age at diagnosis; (**c**) tumour IDH mutation; (**d**) tumour MGMT methylation; (**e**) debulking surgery vs. biopsy alone; (**f**) chemo/radio-therapy after diagnosis.

Overall, the median survival was 9.2 months (IQR 7.9 to 10.3 months) (Figure 2a); the 12- and 24-month survival rates were 40.7% (95% CI 36.3–45.1) and 13.3% (95% CI 10.3–16.6), respectively.

In the univariate analyses of variables known at the time of diagnosis, advancing age was associated with a shorter survival (hazard ratio vs. age < 50: 1.70 [1.26–2.30] for ages 50–59; 3.53 [95% CI, 2.65–4.70] for ages 60–69; 4.82 [95% CI 3.54–6.56] for ages 70+), whereas an *IDH* mutation (HR 0.64 [0.46–0.89]) or *MGMT* promotor methylation (HR 0.80 [0.66–0.97]) were associated with longer survival (Table 2, Figure 2). Interestingly, the improved survival among patients with *MGMT* promoter methylation occurred almost exclusively after 9 months of follow up (Figure 2d). Compared with patients without *MGMT* promotor methylation, patients with methylated promotors had an almost identical risk of death during the first 9 months (HR = 0.98 [95% CI 0.76 to 1.30]), but a greatly reduced risk of death thereafter (HR = 0.62 [95% CI 0.47 to 0.83], *p* for interaction of hazard ratio over time = 0.121). Other tumour characteristics were not significantly associated with survival. In multivariate analyses, age and *MGMT* promoter methylation, but not *IDH* mutation, remained significant predictors of survival.

In further multivariate analyses restricted to the 314 patients in whom adjuvant treatment was known, debulking surgery and type of further treatment were both identified as predictors of survival (Table 3). Debulking surgery was associated with longer survival compared with biopsy only (adjusted HR 0.54 [95% CI 0.41–0.70]), with median survivals of 14.9 vs. 8.0 months in patients with debulking surgery and biopsy, respectively, and 24-month survival rates of 23.4% vs. 4.5%. When compared with no further therapy (median survival 2.0 months), non-standard therapy (median survival 9.2 months, adjusted HR = 0.19 [95% CI 0.13 to 0.29]) and standard therapy (median survival = 16.9 months, adjusted HR = 0.09 [0.06 to 0.13]) were associated with progressively longer survival. Twenty-four month survival rates among patients with no further therapy, non-standard therapy, and standard therapy were 0%, 3.8%, and 30.5%, respectively.

In those who had treatment in the relapsed setting, having further chemotherapy was associated with longer survival [HR 0.39, 95% CI 0.29 to 0.54 (age/sex adjusted)] and having further surgery was associated with longer survival, although this was non-significant [HR 0.77, 95% CI 0.56 to 1.07 (age/sex adjusted)].

## 4. Discussion

This study confirms the major treatment-independent prognostic factors of age and tumour *MGMT* promotor methylation, and supports the role of debulking surgery and chemoradiotherapy in newly diagnosed glioblastoma.

Younger age is consistently recognised as the most significant prognostic variable [4,18,19,20,21,22,23]. In our study, as well as being an independently positive prognostic factor, younger patients were more likely to have debulking surgery rather than biopsy, and were more likely to receive standard rather than non-standard or no therapies; both of which were associated with longer survival.

*MGMT* mediates a DNA repair mechanism, and epigenetic silencing through methylation of the *MGMT* promotor confers a positive prognosis and predicts response to temozolomide (an alkylating agent) in patients with glioblastoma [8,11,18,24,25]. In patients unfit for standard therapy such as the elderly, *MGMT* promotor methylation is frequently used to stratify whether patients should have chemotherapy, an approach that is supported by clinical trials [26,27]. Our study supports these findings, and the apparent divergence of the survival curves after 9 months supports a potential treatment related effect.

In our study, the presence of an *IDH* mutation was associated with longer survival, but was also associated with younger age (49 years with vs. 60 years without). It was not independently associated with survival in adjusted models, although statistical power to detect an association was limited because only 10% of patients had the *IDH* mutation. *IDH* mutation is an early event in tumourgenesis, and *IDH* mutant tumours are considered clinically and genetically distinct from those that are *IDH* wild type. They were first classified separately within the 2016 WHO Classification (as glioblastoma, IDH-mutant) and have been further distinguished as a separate entity within the recent 2021 WHO Classification, which removed the nomenclature glioblastoma entirely, terming them as astrocytoma, IDH-mutant, CNS WHO grade 4 [1,16,28,29]. This is in keeping with the long-held view that they are considered to represent high grade transformation from a lower grade lesion, and most studies and meta-analyses have identified *IDH* mutation as an independently favourable prognostic factor [30,31,32,33,34,35], although more complex interactions between age and molecular groups have been proposed [36].

Our study is limited by the retrospective study design. We were unable to collect and statistically adjust for some known prognostic indicators of survival; as the selection bias of patients to different treatments is so strong, the extent to which survival outcomes are driven by differences in treatments is unclear. Performance status is consistently recognised as an independent prognostic variable [18,19,20,21,22,23]. No standardised performance status was available in the vast majority of our patients and we felt retrospective designation risked unacceptable bias. While resection over biopsy is an accepted prognostic marker and is supported by our study, and it is generally (although not always) agreed that the complete resection is favourable over partial resection, the degree of resection required to offer improved survival remains unestablished [37,38,39]. However, it was not standard practice in our centre to perform post-operative imaging at the time of this study, and so our data cannot address this important research question. We attempted to account for these limitations by performing both treatment-independent and treatment-dependent analyses (Table S2), which both determined *MGMT* promotor methylation and younger age as independent prognostic variables in our cohort.

## 5. Conclusions

In conclusion, in this single-institution retrospective cohort review of 490 consecutively newly diagnosed patients with glioblastoma, IDH-wildtype, CNS WHO grade 4, and astrocytoma, IDH-mutant, CNS WHO grade 4, the median survival from diagnosis was 9.2 months. The median survival in patients who received debulking surgery at diagnosis was 14.9 months vs. 8.0 months in those who had a biopsy only. Following diagnosis, median survival in those treated with standard therapy (radical radiotherapy with temozolomide chemotherapy) was 16.9 months, compared with 9.2 months for those who received other regimens of radiotherapy or chemotherapy, and 2.8 months in those patients who received no subsequent therapy. Multivariate analysis treatment-independent variables at diagnosis identified younger age and tumour *MGMT* promotor methylation to be positive prognostic markers.

## Figures and Tables

**Table 1:** Patient Characteristics.

Patient Characteristics	Percentage (n/N with data)
Age (Mean ± SD)	59.2 ± 13.1
Male	59.8% (293/490)
Female	40.2% (197/490)
*Tumour molecular markers:*	
*IDH* mutation	10.6% (51/482)
*MGMT* promotor methylation	51.3% (234/456)
Loss of *PTEN* locus	45.6% (165/362)
*EGFR* amplification	42.1% (151/359)
1p and 19q LOH	6.1% (15/245)
Debulking surgery	60.0% (294/490)
*Radio-/chemo-therapy*^1^:	
Standard ^2^	56.1% (176/314)
Non-standard	24.8% (78/314)
None	19.1% (60/314)

^1^ Among patients surviving 6 weeks. ^2^ RT 60–65 Gy/30# with TMZ chemotherapy.

**Table 2:** Univariate and multivariate analyses of treatment independent survival characteristics.

Biomarker	Patients Included in Analysis	N	Median Survival with Factor (95% CI)	N	Median Survival without Factor (95% CI)	Unadjusted Hazard Ratio (95% CI)	Final Model ^1^ (95% CI)
Male (vs. Female)	490	293	8.8 (7.2 to 10.2)	229	9.7 (7.9 to 12.5)	1.21 (1.00 to 1.47)	-
*Age:*	490						
<50	110	110	16.7 (14.5 to 20.7)	-	-	1.00	1.00
50–59	110	110	14.0 (11.5 to 16.4)	-	-	1.70 (1.26 to 2.30)	1.67 (1.22 to 2.28)
60–69	155	155	6.1 (4.9 to 7.5)	-	-	3.53 (2.65 to 4.70)	3.54 (2.61 to 4.80)
70+	115	115	5.6 (3.9 to 6.7)	-	-	4.82 (3.54 to 6.56)	5.03 (3.65 to 6.92)
*PTEN* mutation	362	165	9.2 (6.9 to 11.2)	197	10.1 (8.0 to 12.5)	1.14 (0.92 to 1.42)	-
*EGFR* amplification	359	151	9.3 (7.9 to 11.3)	208	9.4 (6.8 to 12.1)	1.07 (0.86 to 1.34)	-
1p and 19q LOH	245	15	14.1 (2.3 to >24)	230	10.5 (8.4 to 12.8)	0.54 (0.29 to 1.00)	-
*MGMT* promotor methylation	456	234	9.4 (7.5 to 11.6)	222	9.1 (7.3 to 10.3)	0.80 (0.66 to 0.97)	0.71 (0.58 to 0.87)
*IDH* mutation	482	51	10.3 (7.7 to 20.1)	431	8.9 (7.5 to 10.1)	0.64 (0.46 to 0.89)	-

^1^ Final model chosen by stepwise variable selection on full data (n = 490) after fully conditional specification multiple imputation.

**Table 3:** Survival characteristics by treatment.

Characteristic	Patients Included in Analysis	Median Survival with Factor (95% CI)	Adjusted Hazard Ratio ^1^ (95% CI)
*Debulking surgery:*	314		
No	113	8.0 (6.7 to 9.7)	1.00
Yes	201	14.9 (13.1 to 16.7)	0.54 (0.41 to 0.70)
*Radio-/chemo-therapy:*	314		
None	176	2.8 (2.3 to 3.4)	1.00
Non-standard	78	9.2 (7.5 to 11.4)	0.19 (0.13 to 0.29)
Standard ^2^	60	16.9 (15.8 to 18.3)	0.09 (0.06 to 0.13)

^1^ Adjusted for MGMT and age, on full data (n = 314), after fully conditional specification multiple imputation. ^2^ RT 60–65 Gy/30# with TMZ chemotherapy.

## Data Availability

The data presented in this study are available on request from the corresponding author.

## Supplementary Materials

The following supporting information can be downloaded at: https://www.mdpi.com/article/10.3390/cancers14133161/s1. Figure S1: Age at diagnosis. Table S1: Age at diagnosis by clinical characteristics. Table S2: Final Survival Model.
