# Supplementary material for: Survival Outcomes and Prognostic Factors in Glioblastoma
Source: Cancers (Basel). 2022 Jun 28;14(13):3161. doi: 10.3390/cancers14133161 (PMC9265012; doi:10.3390/cancers14133161)
Supplement: Supplementary file 1 [file cancers-14-03161-s001.zip › cancers-1746385-supplementary.pdf]

cancers

MDPI

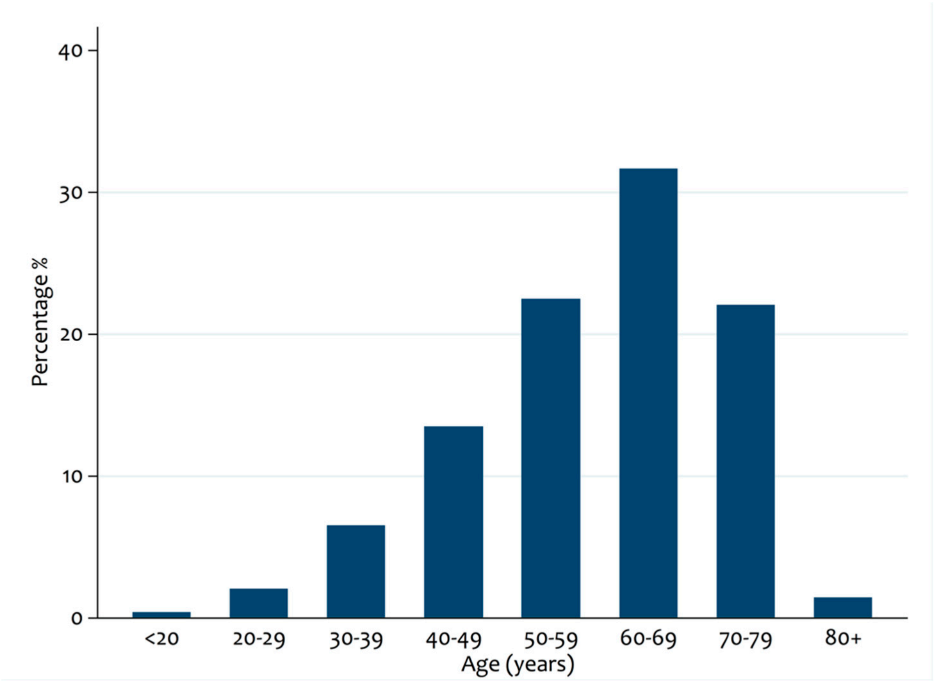

Figure S1. Age at diagnosis.

Table S1. Age at diagnosis by clinical characteristics.

Clinical characteristic	Age in those without factor	Age in those with factor	P-value for association
Male (vs Female)	58.8	59.5	0.561
Tumour molecular markers:			
IDH mutation	60.3	49.4	<0.001
MGMT promotor methylation	58.9	59.7	0.536
Loss of PTEN locus	58.4	59.3	0.530
EGFR amplification	58.1	59.7	0.264
1p and 19q LOH	58.5	50.4	0.023
Debulking surgery	63.3	56.4	<0.001
Radio-/chemo-therapy[1]*:*			
None	54.8	68.8	<0.001
Non-standard	56.6	60.2	
Standard	63.9	52.4	

[1] Amongst patients surviving 6 weeks.

Table S2. Final survival model (n=314).

Characteristic	Adjusted hazard ratio (95% CI)
Debulking:	
No	1.00
Yes	0.54 (0.41 to 0.70)
Radio-/Chemo-therapy:	
None	1.00
Non-standard	0.19 (0.13 to 0.29)
Standard	0.09 (0.06 to 0.13)
Age:	
<50	1.0
50-59	1.26 (0.88 to 1.81)
60-69	3.13 (2.20 to 4.46)
70+	2.09 (1.37 to 3.20)
MGMT promotor methylation:	
No	1.0
Yes	0.63 (0.49 to 0.83)
